# Development of a double monoclonal antibody–based sandwich enzyme-linked immunosorbent assay for detecting canine distemper virus

**DOI:** 10.1007/s00253-020-10997-y

**Published:** 2020-11-07

**Authors:** Yuan Zhang, Gang Xu, Lu Zhang, Jiakai Zhao, Pinpin Ji, Yaning Li, Baoyuan Liu, Jingfei Zhang, Qin Zhao, Yani Sun, En-Min Zhou

**Affiliations:** 1 Department of Preventive Veterinary Medicine, College of Veterinary Medicine, Northwest A&F University, Yangling, 712100 Shaanxi China; 2 Scientific Observing and Experimental Station of Veterinary Pharmacology and Diagnostic Technology, Ministry of Agriculture, Yangling, 712100 Shaanxi China; 3 Xi’an Center for Animal Disease Control and Prevention, Xi’an, 710061 Shaanxi China

**Keywords:** Canine distemper virus, CDV-F protein, Monoclonal antibody, Sandwich ELISA

## Abstract

**Abstract:**

Canine distemper virus (CDV) infection causes mass mortality in diverse carnivore species. For effective virus surveillance, rapid and sensitive assays are needed to detect CDV in field samples. In this study, after BABL/c mice were immunized with recombinant CDV-fusion (F) protein, monoclonal antibodies (mAbs) against recombinant CDV-F protein (designated 1A5, 1A6, and 7D5) were produced using traditional hybridoma cell technology. Next, capture antibody (1A6, 800 ng/well) and horseradish peroxidase (HRP)–conjugated detection antibody (HRP-7D5, 1:100, 500 ng/well) were used in a double monoclonal antibody–based sandwich enzyme-linked immunosorbent assay (ELISA) for CDV detection after optimization of both mAb amounts per well using a checkerboard titration test. Based on sandwich ELISA test results for 120 known CDV-negative samples, the cutoff value for a positive result was set to an OD_450 nm_ value ≥ 0.196. As compared with test results obtained from commercial immune colloidal gold test strips, the low limits of detection for the two assays were revealed to be 100 TCID_50_ per 100 μL. In addition, the sandwich ELISA agreed 100% and 96.4% with commercial immune colloidal gold test strips when testing serum and stool samples. The sandwich ELISA assay provided statistically similar CDV detection. Thus, the sandwich ELISA developed here to detect CDV in fecal and serum samples provided good sensitivity, high specificity, and good reproducibility and should serve as an ideal method for large-scale surveillance of CDV infections in carnivores.

**Key points:**

• *Three CDV mAbs that recognized different epitopes and bound to virion were generated.*

• *The sandwich ELISA based mAbs to detect CDV in fecal and serum samples was developed.*

• *The sandwich ELISA is an ideal method for detecting CDV infections in the field.*

## Introduction

Canine distemper virus (CDV) can infect a broad range of carnivores, including *Canidae*, *Procyonidae*, *Felidae, Mustelidae*, *Mephitidae*, *Ailuridae*, *Viverridae*, *Hyaenidae*, and *Phocidae* (Beineke et al. 2015; Loots et al. 2017). Viral infection can lead to the development of complex clinical signs that include respiratory, gastrointestinal, and neurological symptoms. Generally, the mortality rates of CDV infection for most susceptible animal species have ranged from 30 to 80%, while the corresponding rate in ferrets is 100% (Deem et al. 2000; von Messling et al. 2003). Recently, the diseases caused by CDV infection have even occurred in vaccinated dogs and important economic animal species, including farmed mink, fox, and racoon dog, resulting in serious economic losses (von Messling et al. 2003; Pope et al. 2016). In addition, CDV infections of many endangered animals, including Amur tiger, Ethiopian wolf, and giant panda species, have also been reported (Loots et al. 2017).

CDV, a member of the genus *Morbillivirus* within the family *Paramyxoviridae*, is an enveloped, non-segmented, single-stranded RNA virus (Duque-Valencia et al. 2019; Li et al. 2018a, b). Its 15,690-nt viral genome encodes six open reading frames (ORFs) corresponding to six proteins: nuclear protein (N), phosphoprotein (P), matrix protein (M), fusion protein (F), hemagglutinin protein (H), and large protein (L) (Martinez-Gutierrez and Ruiz-Saenz 2016; Sidhu et al. 1993). Of these proteins, CDV H and F glycoproteins are located on viral particle surfaces and are responsible for virus attachment to host cells and fusion with the host cell membrane (Rendon-Marin et al. 2019). Importantly, several previous studies have shown that the F protein is a highly conserved immunogenic protein and an important antigen for inducing neutralizing antibodies against CDV (David et al. 2019).

Diagnosis of CDV infection is usually based on clinical signs and history. In early stages of CDV infection in unvaccinated puppies, CDV infection is often indistinguishable from other diseases, such as kennel cough. Thus, the rapid and accurate detection is an important measure for controlling disease. Currently, many experimental detection methods have been developed to diagnose CDV infection accurately, such as virus isolation, reverse transcription polymerase chain reaction (RT-PCR), real-time RT-PCR, immunohistochemical detection, and immune colloidal gold test strips (Barben et al. 1999; Cho et al. 2014; Elia et al. 2006; Martella et al. 2007; Nemeth et al. 2018; Soma et al. 2003; Wang et al. 2017). At present, detection of CDV infection has mainly relied on RT-PCR or real-time RT-PCR and testing the CDV viral particles using immune colloidal gold test strips. However, RT-PCR assays can only be carried out in well-equipped laboratories (Kim et al. 2006; Wang et al. 2018), while immune colloidal gold test strip testing is expensive and therefore unsuitable for testing of large numbers of samples. For these reasons, here we developed a sandwich enzyme-linked immunosorbent assay (ELISA) as a sensitive, specific, and easy-to-implement assay for detection of viral antigens that could be suitable for high-throughput testing applications. This sandwich ELISA was designed to detect CDV in serum and fecal samples based on specific binding of monoclonal antibodies (mAbs) to CDV-F protein in this work. This monoclonal antibody–based sandwich ELISA offers good reliability, specificity, simplicity, and reproducibility for clinical detection of CDV.

## Materials and methods

### Cells and viruses

Vero cells and SP2/0 murine myeloma cells were purchased from ATCC and were cultured in Dulbecco’s Modified Eagle’s Medium (DMEM, Life Technologies Corp, USA) supplemented with 10% fetal bovine serum (FBS, Gibco, USA) at 37 °C in 5% CO_2_.

Canine distemper virus (CDV) strain *Onderstepoort* (GenBank number: EU143737.1) was propagated in Vero cells using a viral stock with 50% tissue culture infective dose (TCID_50_) value of 10^5^/mL.

### Expression and purification of recombinant CDV-F protein

The complete gene encoding the CDV-F protein was cloned based on the CDV reference genome sequence (GenBank accession number KP677502) and ligated to pET-28a vector (Novagen USA) by GENEWIZ Company. Next, the positive recombinant plasmid was transformed into *Escherichia coli* (*E. coli*) BL21 (DE3)-competent cells for the expression of CDV-F protein followed by screening of transformants for the desired plasmid construct. Next, the expression of recombinant CDV-F protein in verified transformants was induced by addition of 0.1 mM isopropyl-β-d-thiogalactoside (IPTG); then, bacteria were harvested by centrifugation, lysed by sonication. After centrifugation of the sonicate, the pellet containing inclusion bodies was washed then resuspended in a solution containing 8 M urea in phosphate-buffered saline (PBS, pH 7.2). Finally, the recombinant CDV-F protein was purified via metal affinity chromatography (IMAC) using a Ni-NTA Superflow chelating agarose column according to the manufacturer’s instructions. Expression, purification, and antigenicity of the CDV-F protein were analyzed using SDS-PAGE and Western blot assays. For the Western blot assay, the commercially available dog anti-CDV antibodies (XiNuo Bio-Technology Limited, Changchun, China) were served as the primary antibody.

### Production and characterization of mAbs against CDV-F protein

Four 6-week-old BALB/c mice were purchased from the Experimental Animal Center of Xi’an Jiaotong University and immunized intraperitoneally at 2-week intervals with the purified CDV-F protein (100 μg/mouse) for a total of three inoculations. For the first immunization, the protein was emulsified with an equal volume of Freund’s complete adjuvant. For the other two inoculations, protein was emulsified with Freund’s incomplete adjuvant. One month after the third injection, the titers of antibodies against CDV-F protein in serum samples from the mice were detected by indirect ELISA using the purified recombinant protein CDV-F protein as coating antigen. Before harvesting cells for cell fusions to generate hybridomas, mice were given a final booster injection by tail vein. Five days later, mice were anesthetized with a dose of ketamine and acepromazine (100 mg/kg K + 5 mg/kg A) via intraperitoneal injection then sacrificed by cervical dislocation. Next, spleen cells from immunized mice were fused with SP2/0 murine myeloma cells using a standard polyethylene glycol–mediated fusion method. Hybridoma cell lines secreting antibodies against CDV-F protein were identified using indirect ELISA then positive hybridoma lines were subcloned twice to establish stable clones. Next, mAbs in culture supernatants were purified using Protein G columns according to the manufacturer’s instructions (Jinsite Company, Nanjing, China). Purified mAbs were analyzed by SDS-PAGE, and their concentrations were calculated from spectrophotometrically determined absorbance values based on an absorption coefficient of OD_280nm_/(1.35 mg/mL). Animal experiments were conducted under the guidelines of Animal Care and Use Committee of Northwest Agricultural & Forestry University (NWSUAF, Permit Number: AE189693).

To determine which mAbs bound specifically with the F protein of CDV particles, the purified mAbs were used as primary antibodies for conducting F protein detection in CDV-inoculated Vero cells via immunofluorescence assays (IFAs) (Yahara et al. 2002).

To identify mAbs isotypes, the Mouse Monoclonal Antibody Isotyping Reagents (ISO2) (Sigma) kit was used according to the manufacturer’s instructions.

To determine the spatial relationships of epitopes recognized by the mAbs in this study (Dong et al. 2011; Zhou and Afshar 1999), the purified mAbs were first conjugated to horseradish peroxidase (HRP) using a peroxidase-labeling kit according to the manufacturer’s instructions (Roche Diagnostics, Basel, Switzerland). Next, the HRP-labeled antibody was used in a competitive ELISA that was performed according to the procedure described below.

### Indirect and competitive ELISAs

Indirect ELISA was used to detect the titers of anti-CDV-F protein antibodies in immunized mouse sera and identify the mAbs against CDV-F protein in hybridoma supernatants binding with the antigens in 96-well plates (Nunc). Plates were coated with purified CDV-F protein (200 ng/well) and incubated at 4 °C overnight then blocked with blocking buffer (PBS-T: 0.01 M PBS, pH 7.2, supplemented with 2.5% dried milk (*w*/*v*) and 0.5% Tween-20 (*v*/*v*)) at room temperature (RT) for 1 h. Sera (diluted in blocking buffer) or hybridoma supernatants were added and incubated at RT for 1 h. Anti-mouse IgG (H + L)-HRP (Jackson ImmunoResearch, USA) was then added and incubated at RT for 1 h followed by addition of tetramethylbenzidine (TMB) (A: 205 mM potassium citrate (pH 4.0); B: 41 mM tetramethylbenzidine; A:B (*v*/*v*) = 39:1) to induce the colorimetric reaction. Finally, the OD_450 nm_ values were read using an automated ELISA plate reader (Bio-Rad, USA) after the reaction was stopped by the addition of 3 M H_2_SO_4_.

Competitive ELISA was used to determine the spatial relationships of epitopes recognized by the mAbs developed in this study. First, the titers of HRP-conjugated mAbs (HRP-mAbs) at dilutions of 10^−1^ to 10^−4^ were measured using direct ELISA. Dilutions of HRP-mAbs used in competitive ELISAs were selected based on OD_450 nm_ values of approximately 1 as determined previously by direct ELISA. Using purified CDV-F protein as coating antigen for the competitive ELISA, the mAbs were used as competitors then HRP-mAbs were sequentially added into wells. After color development of reactions proceeded, reactions were stopped and OD_450 nm_ values were recorded. Maximal binding without inhibition was found when HRP-mAbs were added without competitors. The percentage of inhibition was calculated using the formula: 100 × [1 − (OD_450 nm_ of HRP-mAbs and mAb)/(OD_450 nm_ of HRP-mAb)]. Competitive binding of a given HRP-mAb was considered inhibited if binding was decreased by 40% or more.

### Development of double monoclonal antibody–based sandwich ELISA

To develop the sandwich ELISA, the pair of mAbs was selected as capture and detection reagents. An orthogonal experiment using these mAbs was designed as follows: first, the different mAbs (800 ng/well) were used to coat wells of ELISA plates as capture antibodies and incubated at 4 °C overnight. After plates were blocked and washed, CDV viral stock (positive control, P) or culture supernatant of normal Vero cells (negative control, N) were added. After washing again, equivalent amounts of individual HRP-mAbs were added to separate wells. After a final wash, TMB was added to induce the colorimetric reaction and OD_450 nm_ values were read after reactions were stopped. Selection of the best pair of mAbs was based on the highest P/N value.

Second, optimal amounts of capture mAb and detection HRP-mAb for the sandwich ELISA were determined using a checkerboard titration method (Chen et al. 2016; Liu et al. 2014). Different amounts of capture mAb (100, 200, 400, 800, and 1000 ng/well) and different dilutions of HRP-mAb (1:1, 1:10, 1:100, 1:1000, and 1:10,000) were used in the sandwich ELISA. The CDV viral stock (100 μL/well) served as positive control and the same volume of normal Vero cell culture supernatant served as negative control. Optimal amounts of capture mAb and detection HRP-mAb were determined based on the highest P/N value.

### Validation of the sandwich ELISA

To determine the cutoff value for positive results of the sandwich ELISA developed here, the 120 negative samples, including cloacal swabs (*n* = 25) from pet dogs, supernatants from normal Vero cell cultures (*n* = 15) and serum and fecal samples from healthy pet dogs (*n* = 80) were tested. Clinical samples were collected and used for evaluating the sandwich ELISA with the consent of the animal owners. Cloacal swabs and fecal samples were suspended in 100–200 μL PBS, then ground and centrifuged. The cutoff value for interpretation of positive and negative results was based on the mean OD_450 nm_ value obtained from values of 120 negative samples plus 3 standard deviations (SD) to ensure the cutoff value would provide 99% confidence for discriminating between positive and negative results.

To determine the viral detection limit of the sandwich ELISA developed here, different amounts (from 10 TCID_50_ to 10^4^ TCID_50_) of CDV viral stocks were tested using the sandwich ELISA and compared to results obtained for known amounts of virus tested using commercial immune colloidal gold test strips (Beijing Anheal Laboratories Company, Beijing, China).

To determine the specificity of the sandwich ELISA, other canine disease viruses, including canine parvovirus (CPV), canine infectious hepatitis virus (ICHV), and canine parainfluenza virus (CPIV), were also tested using the assay.

The reproducibility of the sandwich ELISA was evaluated by testing 3 positive (CDV viral stock) and 3 negative samples (supernatants from normal Vero cell cultures). The coefficient of variation (CV) was used to evaluate the inter-assay variation (between plates) and the intra-assay variation (within a plate). The six samples were tested using three different plates tested on different occasions to determine the inter-assay CV, while three replicates within each plate were used to calculate the intra-assay CV.

### Comparison between sandwich ELISA and commercial immune colloidal gold test strips

To determine the agreement rate of CDV detection results obtained for clinical samples using the sandwich ELISA developed here and commercial immune colloidal gold test strips, 43 serum samples (13 panda and 30 dog specimens) and 56 clinical fecal samples from dogs were tested using both methods. The result of the agreement rate between the two test methods was calculated using EXCEL software based on test results for each specimen.

### Statistical analysis

Kappa index values were calculated to estimate the level of coincidence of results obtained via sandwich ELISA versus commercial immune colloidal gold test strips. These calculations were performed using SPSS software (version 20).

## Results

### Production of recombinant CDV-F protein

**Fig. 1:**
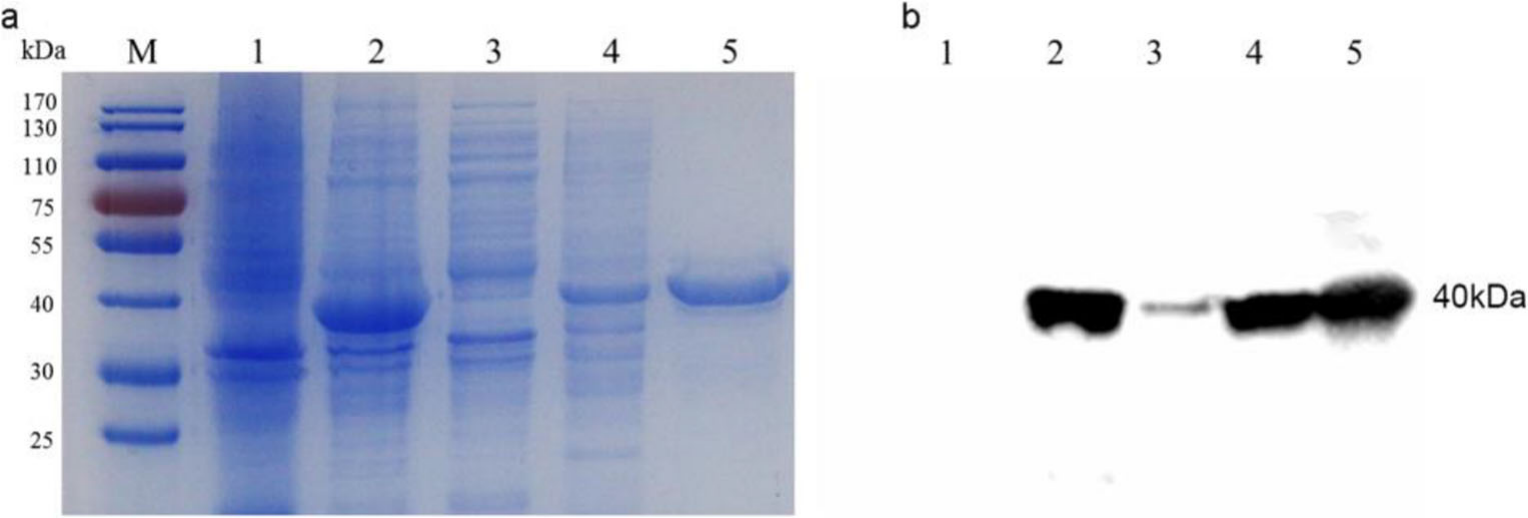
Expression, purification, and identification of recombinant CDV-F protein with the bacterial system. **a** SDS-PAGE analysis. M: protein marker; lane 1: pET-28a vector control; lane 2: bacterial lysates of the CDV-F; lane 3: solution protein; lane 4: inclusion body; lane 5: purified protein. The relative molecular masses of CDV-F protein was about 40 kDa. **b** Antigenic analysis of Western blot, lanes 1–5: same as **a**, reaction with high immune antibody against CDV from the dog

To produce mAbs against CDV-F protein, the recombinant CDV-F protein was expressed in an *E. coli* system to yield inclusion bodies. The SDS-PAGE analysis of inclusion body proteins indicated the presence of CDV-F protein with a molecular weight of approximately 40 kDa, as expected (Fig. 1a). Next, the F protein was purified using a Ni-NTA resin column; then, the SDS-PAGE analysis was conducted and demonstrated the presence of highly pure recombinant F protein (Fig. 1a). This protein was subsequently shown via Western blot analysis to possess antigenic epitopes that bound to anti-CDV antibody from dogs possessing strong immunity to CDV (Fig. 1b). Therefore, the purified CDV-F protein was suitable for use as an immunizing antigen to generate mAbs in BALB/c mice.

### Production and characterization of mAbs against CDV-F protein

**Fig. 2:**
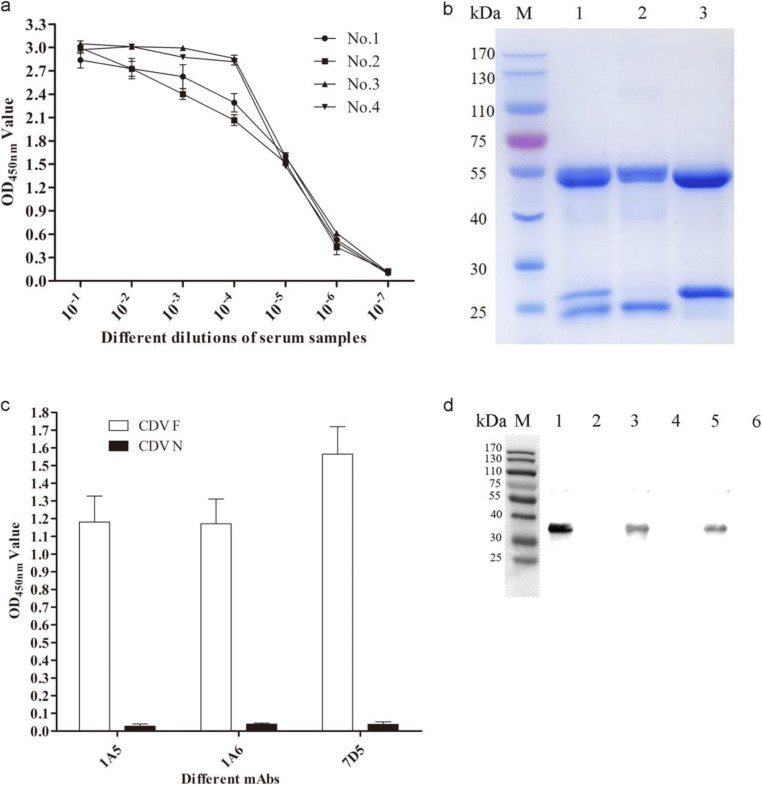
Screening, purification, and characterization the mAbs against the CDV-F protein. **a** Titers of antibodies against CDV-F protein in the sera from the BABL/c mice after the third immunization. **b** SDS-PAGE analysis of purified mAbs 1A5, 1A6, and 7D5. Specific reactions between the 3 screened mAbs and CDV-F protein using indirect **c** ELISA and **d** Western blot. **d** M: protein marker; lanes 1, 3, and 5: purified mAbs 1A5, 1A6, and 7D5 reacted with CDV-F protein, respectively; lanes 2, 4, and 6: purified mAbs 1A5, 1A6, and 7D5 not reacted with CDV-N protein, respectively

Four BABL/c mice were immunized with purified recombinant CDV-F protein. After three immunizations, the antibody titers of serum samples collected from four immunized mice were all 1:10^5^, suggesting that an immune response to recombinant CDV-F protein had been induced in each mouse (Fig. 2a). Next, cells from one randomly selected immunized mouse were used to generate mAbs via methods based on traditional hybridoma technology.

**Fig. 3:**
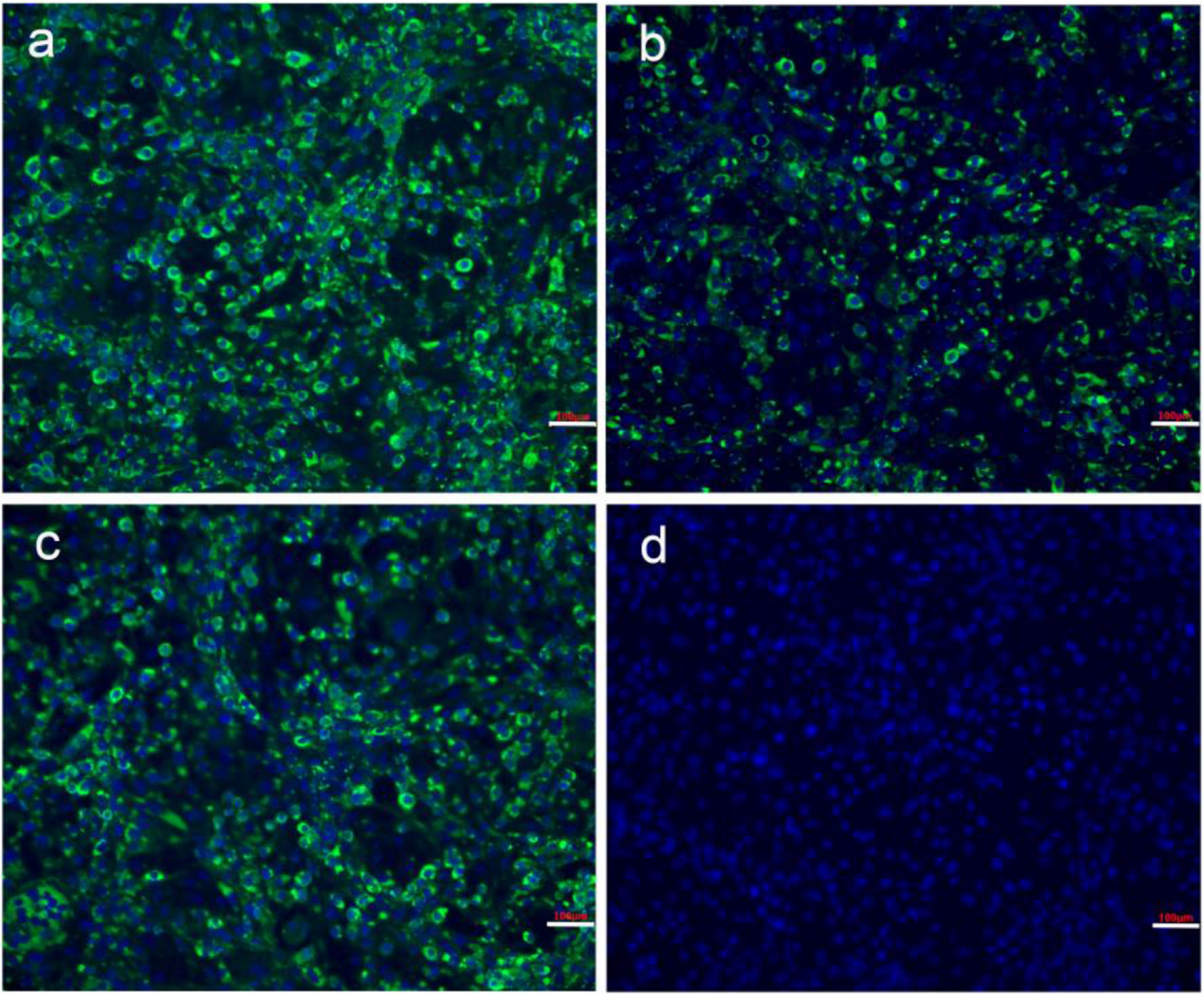
Immunofluorescence assay of three mAbs binding with CDV-F protein in the CDV-infected Vero cells. The CDV-F protein was detected using mAbs and FITC-conjugated goat anti-mouse IgG. **a** 1A5 binding with CDV-F protein. **b** 1A6 binding with CDV-F protein. **c** 7D5 binding with CDV-F protein. **d** Negative control using the mouse IgG as the primary antibody

Three mAbs designated 1A5, 1A6, and 7D5 were successfully produced that subsequently were shown to possess IgG2, IgG2, and IgG1 isotypes, respectively. After concentration followed by purification over a goat anti-mouse IgG affinity column, the mAbs 1A5, 1A6, and 7D5 were successfully purified (Fig. 2b) and had concentrations of 1.31 mg/mL, 1.05 mg/mL, and 1.25 mg/L, respectively. Indirect ELISA and Western blot analysis results showed that these three mAbs specifically reacted with recombinant CDV-F protein and not with recombinant CDV N protein expressed using the same vector and bacterial system (Fig. 2c and d). In addition, IFA results showed that each of the three mAbs could bind to F protein present in CDV-infected Vero cells, suggesting all three mAbs recognized the F protein of CDV particles (Fig. 3).

**Fig. 4:**
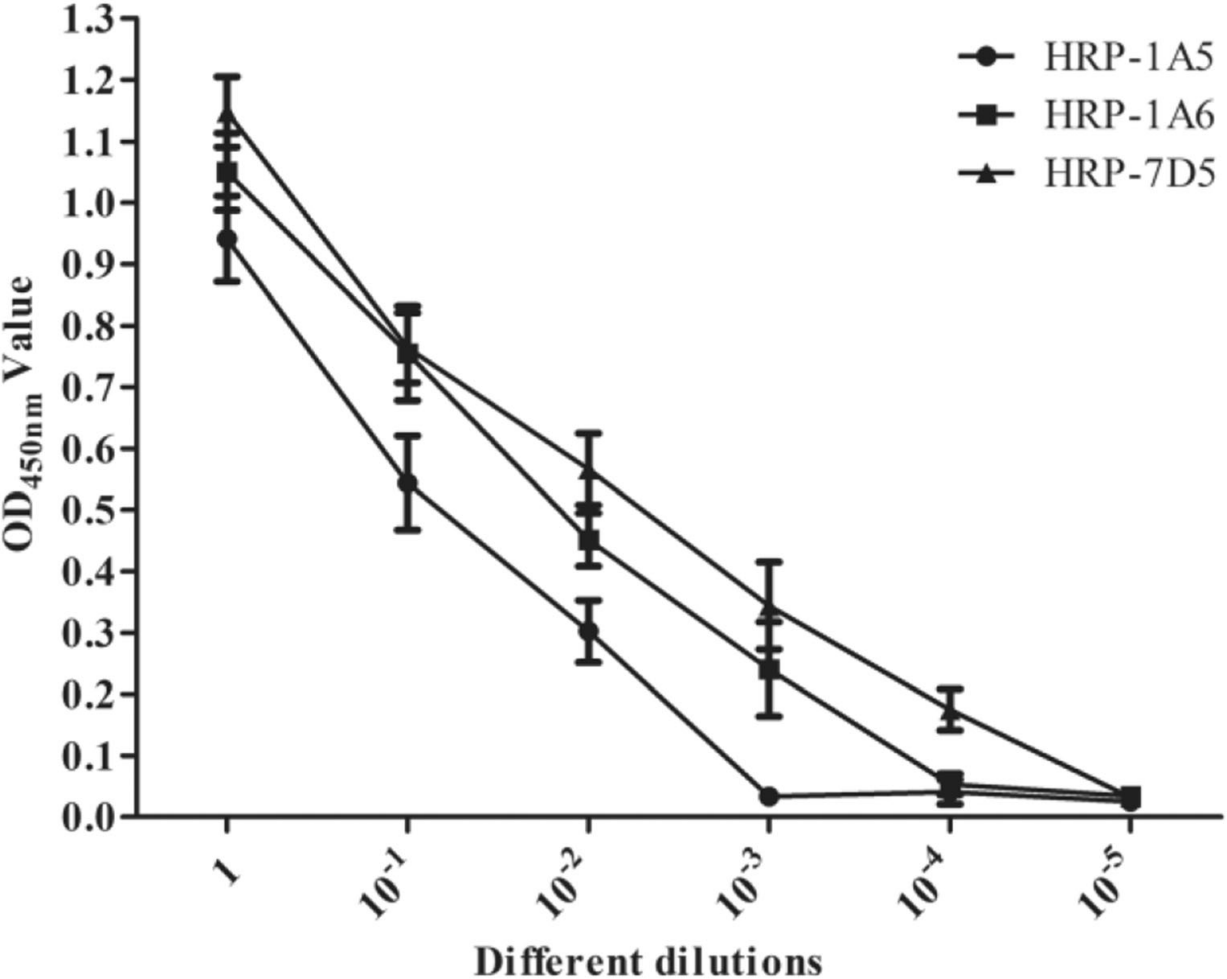
Analysis of the titers of three HRP-labeled mAbs (HRP-1A5, HRP-1A6, and HRP-7D5) to detect CDV-F using direct ELISA. The three HRP-mAbs in a dilution range of 10^0^ to 10^−5^ were tested for reaction with the CDV-F protein in the direct ELISA

After the three mAbs were labeled with HRP, the titers of HRP-1A5, HRP-1A6, and HRP-7D5 were determined to be 10^−2^, 10^−3^, and 10^−4^, respectively, via direct ELISA (Fig. 4). Next, the competitive ELISA results showed that addition of IA5 inhibited HRP-1A5 binding to CDV-F protein by 86.21%, while binding was not inhibited by 1A6 and 7D5, as evidenced by low percent inhibition (PI) values of 8.03% and 10.15%, respectively (Table 1). And binding of HRP-1A6 to CDV-F protein was inhibited by unlabeled 1A6, as evidenced by the resulting PI value of 90.12%, but not by 1A5 and 7D5, as evidenced by low respective PI values of 7.32% and 12.09% (Table 1). These results indicate that the three mAbs, 1A5, 1A6, and 7D5, recognize different epitopes within the CDV-F protein.

**Table 1:** Binding inhabitation of the three labeled mAbs to the recombinant CDV-F protein

HRP-labeled mAbs	Percent inhibition (PI) value (%)		
	1A5	1A6	7D5
HRP-1A5	86.21	8.03	10.15
HRP-1A6	7.32	90.12	12.09
HRP-7D5	9.12	8.41	84.37

### Development of the monoclonal antibody–based sandwich ELISA

To select the best pair of mAbs for use in the sandwich ELISA, the mAbs 1A5, 1A6, and 7D5 were tested separately as capture antibodies and HRP-1A5, HRP-1A6, and HRP-7D5 were tested separately as detection antibodies. Results showed that the P/N value (10.736) was highest for mAb pair 1A6 and HRP-7D5 (Table 2).

**Table 2:** Optimization of the best pair of antibodies for developing the sandwich ELISA

Capture antibody	Sample	Detection antibody		
		HRP-1A5	HRP-1A6	HRP-7D5
1A5	P	-	0.876	0.756
	N	-	0.123	0.088
	P/N	-	7.121	8.617
1A6	P	0.801	-	*1.121*
	N	0.130	-	*0.104*
	P/N	6.162	-	*10.736*
7D5	P	0.901	0.765	-
	N	0.129	0.119	-
	P/N	6.969	6.407	-

Mabs 1A5,1A6 and 7D5 were used as the capture antibody and 1A5-HRP, 1A6-HRP and 7D5-HRP as the detection antibody. CDV viral stock was as the positive control (P) and culture supernatant of normal Vero cells as the negative control (N). Italic represents the best conditions

To optimize amounts of mAb 1A6 and 7D5-HRP for the ELISA, a checkerboard titration assay was used to demonstrate that the optimal amount of mAb 1A6 protein was 800 ng/well and the optimal dilution of 7D5-HRP was 1:10^2^ (500 μg/mL), as shown by the highest P/N value (11.45) obtained using these conditions (Table 3).

**Table 3:** Optimization of the amount of capture mAb 1A6 and dilution of detection antibody HRP-7D5 for developing the sandwich ELISA

Different amounts of 1A6 (ng/well)	Samples	Different dilutions of HRP-7D5			
		1:10	1:10^2^	1:10^3^	1:10^4^
200	P	0.801	0.825	0.432	0.214
	N	0.128	0.094	0.068	0.075
	P/N	6.253	8.776	6.326	2.834
400	P	0.867	0.901	0.567	0.354
	N	0.122	0.091	0.075	0.104
	P/N	7.123	9.876	7.523	3.738
800	P	1.023	*1.123*	0.735	0.358
	N	0.139	*0.098*	0.100	0.088
	P/N	7.345	*11.45*	7.362	4.043
1600	P	1.121	0.987	0.632	0.441
	N	0.144	0.105	0.104	0.098
	P/N	7.762	9.362	6.102	4.501

CDV viral stock was as the positive control (P) and culture supernatant of normal Vero cells the negative control (N). Italic represents the best conditions

After conditions were optimized, the sandwich ELISA was performed as follows: first, the 96-well plate was coated with 800 ng/well of mAb 1A6 in PBS buffer and incubated at 4 °C overnight (Table 3). The next day, plate wells were blocked with blocking buffer (300 μL/well) for 1 h at RT then washed three times with the PBS-T (300 μL/well). Next, test samples (100 μL) were added to wells and plates were incubated for 1 h at RT. After three washes, HRP-7D5 (diluted 1:100 to 500 μg/mL, 100 μL/well) was added then plates were incubated for 1 h at RT (Table 3). After a final wash, TMB (100 μL/well) was added to induce the colorimetric reaction and OD_450 nm_ values were read using an automated ELISA plate reader after the reaction was stopped with 3 M H_2_SO_4_ (50 μL/well).

### Cutoff value of the developed sandwich ELISA

Using the abovementioned procedures, the 120 negative samples were tested using the sandwich ELISA, yielding an average OD_450 nm_ value of 0.121 with SD of 0.025. The cutoff value of the sandwich ELISA was calculated to be 0.196 (0.121 ± 3 SD). Therefore, CDV detection results obtained via sandwich ELISA with OD_450 nm_ values ≥ 0.196 were scored positive for CDV.

### Limitations, specificity, and reproducibility of the sandwich ELISA

**Fig. 5:**
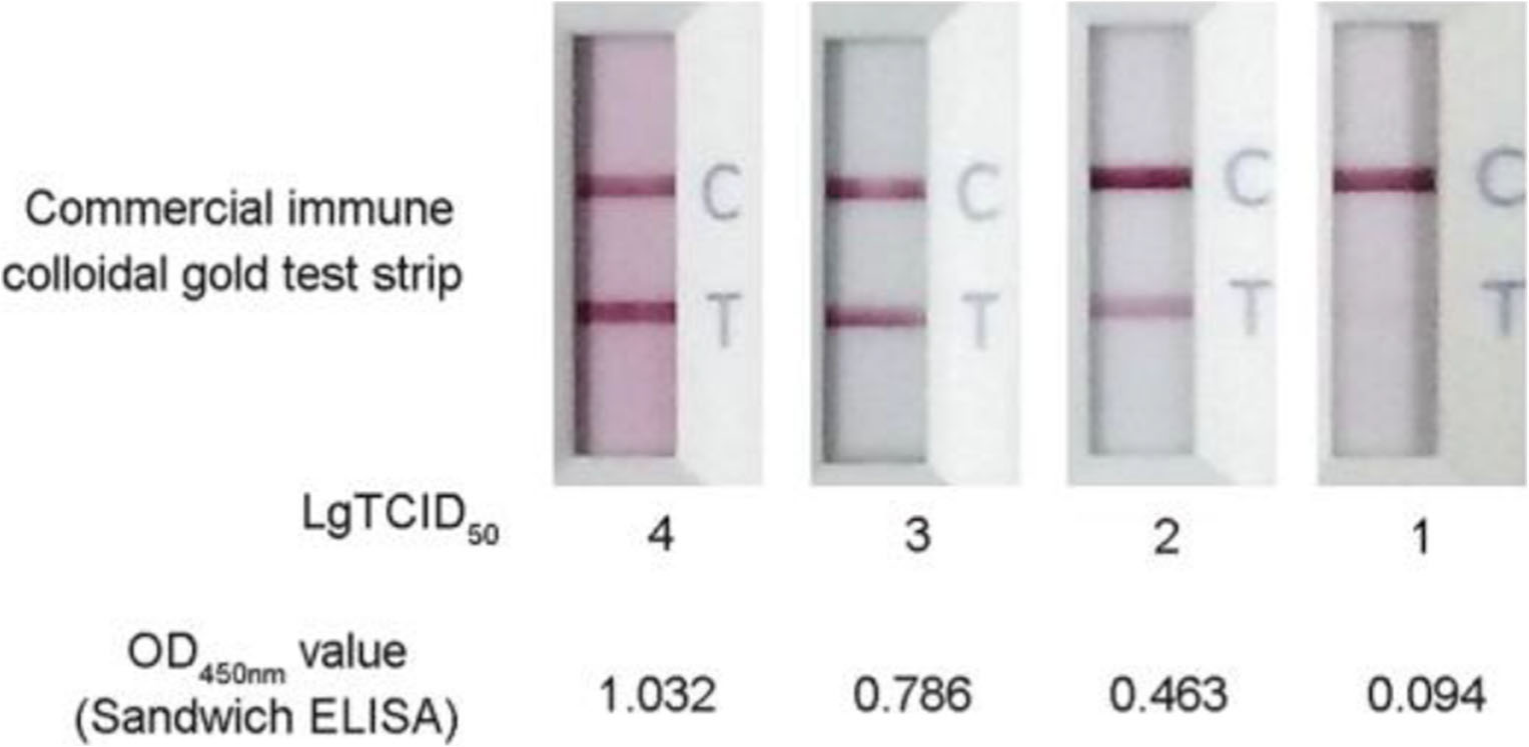
Determination of the limitation of the developed monoclonal antibody–based sandwich ELISA to detect CDV. The different amounts of CDV stock (TCID_50_) were detected by both the developed sandwich ELISA and commercial immune colloidal gold test strip

For determining the lowest detection limit of the sandwich ELISA, different amounts of CDV viral stock were tested using the assay. The results showed that for amounts of CDV stock above 100 TCID_50_ per 100 μL, the OD_450 nm_ values of the sandwich ELISA were all greater than 0.196, indicating that the minimum amount of CDV detected by the assay was 100 TCID_50_ per 100 μL (Fig. 5). Meanwhile, test results obtained for various amounts of CDV using the commercial immune colloidal gold test strip reflected a similar low limit of detection as that obtained using sandwich ELISA (Fig. 5).

To determine the specificity or sandwich ELISA cross-reactivity toward other canine disease viruses, three viruses (CPV, ICHV, and CPIV) were tested via the CDV sandwich ELISA, yielding OD_450 nm_ values of 0.034–0.118, as compared to CDV detection values of 0.46–1.23 for similar quantities of virus. Thus, these results demonstrate that the sandwich ELISA is specific for CDV detection.

By testing three positive and three negative samples in triplicate, intra-assay CV values for the OD_450 nm_ values were observed to range from 3.56 to 8.37%, with a median value of 6.13%. When the six samples were tested in triplicate in three different plates at different times, the inter-assay CV of OD_450 nm_ values ranged from 4.53 to 9.13%, with a median value of 7.37%. These data indicate that the monoclonal antibody–based sandwich ELISA developed here for CDV detection exhibits good reproducibility.

### Agreement between the sandwich ELISA and commercial immune colloidal gold test strip assay

A total of 43 serum samples (from 13 pandas and 30 dogs) and 56 clinical dog fecal samples were tested for CDV via both the sandwich ELISA and commercial immune colloidal gold test strips. Subsequently, sandwich ELISA results exhibited 100% agreement with commercial immune colloidal gold test strip results for CDV detection in serum samples and 96.4% for CDV detection in clinical dog fecal samples (Table 4). Moreover, statistical analysis showed a high level of agreement between results obtained using sandwich ELISA and commercial immune colloidal gold test strip (kappa coefficient = 0.742).

**Table 4:** Comparisons of the developed sandwich ELISA with the commercial immune colloidal gold test trip by detecting serum and fecal samples

Samples	Species	Number	Sandwich ELISA	Commercial immune colloidal gold test		Agreement (%)	Kappa value
				+	–		
Sera	Giant panda	13	–	0	13	100	0.742
	Dog	6	+	6	0		
		24	–	0	24		
Feces	Dog	24	+	22	2	96.4	
		32	–	0	32		

## Discussion

CDV infection is a highly contagious disease that affects domestic dogs, wild animals, endangered species and some important economic animals. Rapid and sensitive laboratory and field tests for CDV infection diagnosis are essential in disease control. Sandwich ELISA methods based on monoclonal antibody have been shown to have many advantages, such as good specificity, high sensitivity, easy standardization, easy mass production, and suitable for high-throughput testing of clinical samples. For these reasons, sandwich ELISA have been applied to the detection of various viruses (Luo et al. 2020; Shao et al. 2019). At present, CDV detection has been limited to the diagnosis of clinical symptoms, serological detection, electron microscopy, and molecular biological test methods. Out of which, the virus isolation and immunohistochemistry detection methods are technically complex and cumbersome. RT-PCR and real-time PCR are widely used for CDV detection, due to their exceptional sensitivity and accuracy (Brown et al. 2020; Jin et al. 2017; Li et al. 2018a, b). However, the two assays were also cumbersome and needed to extract the viral RNA from the samples. Meanwhile, for the RT-PCR and real-time PCR, the professional lab was needed to prevent nucleic acid contamination. In addition, the immune colloidal gold test strip has also been widely used because of its convenience. But the high cost of it, which cost about 2.7–4.4 USD per sample, is already becoming a major burden for the detection of a large number of samples. In contrast, the ELISA is more economical in field testing. Here, the developed sandwich ELISA in the study only cost approximately 0.29 USD per sample, which is much lower than the immune colloidal gold test strip.

CDV-F protein is the viral structural protein that constitutes viral surface protuberances (Wang et al. 2019). Notably, CDV-F protein epitopes have been shown to be the most highly conserved epitopes among different CDV isolates. Therefore, the CDV-F protein was used in this study as the target antigen for preparation of monoclonal antibodies for the sandwich ELISA developed here. Subsequently, all three mAbs obtained in this work were of the IgG subtype, which facilitated further purification. These antibodies were found to be highly specific for CDV-F and thus did not bind appreciably to other CDV proteins. In addition, the three mAbs recognized different F protein epitopes and their spatial conformations were unaffected by HRP labeling, as shown by competitive ELISA. Thus, these results collectively suggest that the CDV-F protein–based sandwich ELISA developed here may be used to detect diverse CDV isolates (Plattet et al. 2007; Wang et al. 2019); in the future, we plan to test numerous CDV isolates to assess whether this assay has universal applicability for detection of diverse CDV isolates.

In this study, mAbs were produced and paired for use in a robust sandwich ELISA for detecting CDV in clinical samples. This detection method provides good specificity for CDV with no observed cross-reactions with other pathogenic canine viruses. Due to the high specificities of mAbs developed here for CDV, the limit of detection of the ELISA approached 100 TCID_50_/mL of CDV, the same limit of detection obtained using commercial immune colloidal gold test strips. Analysis of results between the sandwich ELISA and commercial immune colloidal gold test strips for CDV infection from serum and fecal samples revealed high agreement (kappa coefficient = 0.742) for efficient detection of CDV antigen in clinical samples, highlighting the feasibility of the sandwich ELISA developed here for clinical use.

CDV infection is a growing concern for farmers breeding important economic animals in China, due to steadily increasing numbers of vulnerable farmed mink, ferret, and racoon dogs. Importantly, a canine distemper-like disease has been observed in vaccinated farmed minks, foxes, and raccoon dogs in Shandong, Liaoning, Hebei, and Heilongjiang provinces in northeastern China (Tao et al. 2020). The double monoclonal antibody–based sandwich ELISA established here is easy to conduct and adaptable for high-throughput applications for surveillance of CDV outbreaks in farmed minks, ferrets, and racoon dogs. In the future, testing of numerous clinical samples from such farms will require high-throughput assays to monitor viral infections in the field.

In conclusion, three mAbs specific for CDV-F protein, 1A5, 1A6, and 7D5, were generated in this work using traditional hybridoma cell technology. All three mAbs could bind to the F protein of native CDV particles and thus were suitable for use in a double monoclonal antibody–based sandwich ELISA to detect CDV. Subsequently, mAbs 1A6 and 7D5 were shown to be most suitable for this purpose due to their high sensitivity and high specificity and reproducibility for CDV antigen detection. Therefore, we believe that the sandwich ELISA developed in this work will likely be ideal for surveillance efforts to detect CDV infections in the field.
